# Unravelling influenza correlates of protection: lessons from human A/H1N1 Challenge

**DOI:** 10.1128/mbio.00064-24

**Published:** 2024-03-28

**Authors:** Rebecca Jane Cox, Rishi Pathirana

**Affiliations:** 1 Department of Clinical Science, The Influenza Centre, University of Bergen, Bergen, Norway; 2 Department of Microbiology, Haukeland University Hopsital, Bergen, Norway; McMaster University, Hamilton, Ontario, Canada

**Keywords:** correlate of protection, mucosal immunity, antibody

## Abstract

Mucosal immunity is important in protecting from upper respiratory tract influenza infection. Human challenge provides a unique model to define correlates of protection with baseline immune responses being correlated to the quantity and length of viral shedding and clinical outcomes. Here, we discuss recent work on mucosal and systemic correlates of protection (R. Bean, L. T. Giurgea, A. Han, L. Czajkowski, et al., mBio 15:e02372-23, 2024, https://doi.org/10.1128/mbio.02372-23) and place it in the context of previous work on mucosal immunity. We also discuss the importance of standardized assays to allow global comparison of relevant immune responses in defining correlates of protection. Correlates of protection are important for designing next-generation broadly protective influenza vaccines.

## COMMENTARY

Understanding the correlates of protection (COP) is paramount for designing broadly protective influenza vaccines, as currently licensed influenza vaccines are only moderately effective. The human challenge model can play an important role in defining COP. Indeed, seminal human challenge studies from Hobson et al. showed an inverse correlation between protection from infection and antibodies to the major surface glycoprotein hemagglutinin (HA), crucial for viral entry (1). A protective hemagglutination inhibition (HI) titer from infection was defined as 1/18 to 1/36. Interestingly, Hobson et al. also noted some seronegative individuals were also protected from infection, the first indication that protection is multifaceted. Recent research highlights the importance of multifaceted COP, including T-cell immunity, systemic antibodies to neuraminidase (NA) and the HA stalk, and mucosal antibodies, in conferring protection (2). Moreover, the COVID-19 pandemic has highlighted the importance of mucosal immunity, particularly nasal IgA, in preventing SARS-CoV-2 infection especially from variants and the need for improved understanding and methods for measuring mucosal immunity.

Bean et al. aimed to identify mucosal COP in vaccinated and unvaccinated healthy volunteers in human influenza A/H1N1 challenge studies (3). They describe the differences in mucosal and systemic immune responses after immunization with inactivated influenza vaccine (IIV) followed by influenza human challenge to that of infection with challenge alone. For correlates, the definition of protection is important, be it sterilizing immunity to prevent infection of the upper respiratory tract or reduction in viral load and/or period of shedding, or reduced symptom scores. In this commentary, we discuss the insights gained from these human challenge studies and how to apply knowledge in vaccine design.

The gold standard for understanding the true protective efficacy of influenza vaccines lies in human challenge studies as large-scale efficacy trials are costly and difficult to implement. Challenging vaccinated individuals allows researchers to observe not only symptomatic outcomes but also the dynamics of viral shedding and correlate these to pre-challenge (baseline) immune responses. The Bean et al. study with an updated A/H1N1 human challenge virus has provided a unique opportunity to investigate protective immunity, shedding light on the real-world COP which will aid in the development of improved influenza vaccines.

Inactivated influenza vaccines (IIV) have long been a cornerstone in influenza prophylaxis. IIVs require annual updating to match circulating and vaccine strains to keep pace with the rapid antigenic drift. IIVs are standardized by their HA content, with NA only needing to be present.

Bean et al. showed that IIV predominantly induced antibodies targeting the HA and HA stalk, while stimulating low levels of antibodies against NA. This antibody response was associated with a modest reduction in infection rates following influenza challenge.

Vaccinated subjects had a significantly lower number of days of shedding and symptoms, as well as a lower total number of symptoms. Identification of new COPs for influenza has been a challenge owing to the multifaceted nature of the protective immune response that involves the systemic and mucosal immune responses. Bean et al. showed that pre-vaccination HI titers were the most predictive COP when viral shedding and clinical outcomes were combined, while NA inhibition titers and HA IgG levels only predicted the duration of viral shedding. Lower pre-challenge antibodies were associated with longer periods of viral shedding. This study also confirmed the compartmentalized nature of the influenza vaccine-induced immune responses, where there was no correlation between higher pre-challenge systemic and mucosal antibody responses. The authors also showed that IIV did not induce mucosal antibody responses, while virus challenge resulted in a transient mucosal IgA response, peaking at day 7. Consistent with these observations, we have previously reported the presence of influenza-specific antibody-secreting B cells (ASC) in the adult nasal mucosa, which were not boosted after IIV vaccination (4). Age is also an important factor in understanding COP and lessons can be learnt in children where previous priming can be evaluated. We have earlier shown that previous influenza virus infection is important for the induction of tonsillar IgG, IgA, and IgM ASC in children after IIV, whereas IgM is only induced in unprimed children (5). We have also extensively studied mucosal responses elicited after live attenuated influenza vaccines (LAIV), which remains an excellent model for assessing influenza infection. LAIV was able to induce a strong multifaceted immune response, characterized by follicular helper T cell responses in tonsils and systemic antibody responses in children with low salivary IgA levels, but not in adults with high concentrations of local IgA (6). However, serum antibody responses were not a reliable correlate for immunity for LAIV due to the multifaceted nature of the immune response. Lessons learned from recent transformative vaccine technologies provide new opportunities to understand the complex mechanisms of influenza-induced immunity. For example, the mRNA COVID-19 vaccines induced strong mucosal IgA responses in previously infected individuals (7) providing new insights into the induction of mucosal immunity via parenteral vaccination.

Another challenge in the field is the large variation in immunological assays between laboratories reducing the comparability between immunological endpoints. There is a need for standardized methods of measuring mucosal immunity which allow repeated reproducible sampling of the respiratory tract to better define mucosal correlates of immunity. In Bean’s study, nasal synthetic absorbent material (SAM) strips were collected every other day from 1 to 7 days, whereas nasal washes were collected daily although nasal washes have the problem of reliable collection of adequate quality and volume. We have previously described the considerable effort that was invested in the EU-funded Flucop project where academics, public health institutions, and industry worked together to standardize and harmonize assays that measure influenza COP. The standard operating protocols for HI, NAI, microneutralization (MN), and T-cellular assays, together with accompanying training videos are freely available (8–13). Implementation of standardized assays is critical for comparative analysis of immunological and challenge studies across laboratories and for accelerating vaccine development.

The ongoing evolution of influenza viruses and annual vaccine updates pose continuous hurdles in predicting vaccine efficacy and real-world effectiveness. Despite the wealth of knowledge gained from human immunology studies, human challenge studies remain critical for answering questions about true correlates of protection.
